# Examining the Impact of the COVID-19 Pandemic on Psychosocial Outcomes across Age: A Stress and Coping Framework

**DOI:** 10.1093/geroni/igaa057.3458

**Published:** 2020-12-16

**Authors:** Jillian Minahan, Francesca Falzarano, Neshat Yazdani, Karen Siedlecki

**Affiliations:** 1 Fordham University, New Providence, New Jersey, United States; 2 Weill Cornell Medicine, New York, New York, United States; 3 Fordham University, Bronx, New York, United States; 4 Fordham University, New York, New York, United States

## Abstract

The emergence of COVID-19 and the measures implemented to curb its spread are anticipated to have long-term implications for mental health. Older adults may be at increased risk for adverse mental health outcomes as opportunities to remain socially connected have diminished. Further research is needed to better understand the impact of pandemic-related stress on mental health. Utilizing the stress and coping framework, the purpose of this study is three-fold: 1) to examine the influences of COVID-19-related stress on depression, anxiety, and loneliness, 2) to assess the mediating role of coping style and social support, and 3) to investigate whether these relationships vary across age. Participants (N = 1,318) between the ages of 18-92 years completed an online survey, assessing pandemic-related stress, mental health, social support, coping, and their experiences with social distancing, during the initial implementation of social distancing measures in the United States. Stress, social support, and coping style were related to psychosocial outcomes. Results suggested that avoidant coping mediated the relationship between pandemic-related stress and psychosocial outcomes, particularly depression. Avoidant coping more strongly mediated the relationship between stress and depression in younger adults compared to older adults. Results were consistent with the stress and coping framework and recent work highlighting the older adults’ resilience during the COVID-19 pandemic. Findings highlight the associations between positive coping behaviors and psychosocial well-being and indicate that older adults may use unique adaptive mechanisms to preserve well-being during the COVID-19 pandemic.

